# Overcoming acquired resistance to cetuximab by dual targeting HER family receptors with antibody-based therapy

**DOI:** 10.1186/1476-4598-13-242

**Published:** 2014-10-24

**Authors:** Mari Iida, Toni M Brand, Megan M Starr, Evan J Huppert, Neha Luthar, Harsh Bahrar, John P Coan, Hannah E Pearson, Ravi Salgia, Deric L Wheeler

**Affiliations:** Department of Human Oncology, University of Wisconsin School of Medicine and Public Health, Wisconsin Institute for Medical Research, 1111 Highland Ave., Madison, WI 53705 USA; Division of Hematology/Oncology, Department of Medicine, University of Chicago, Chicago, IL 60637 USA

**Keywords:** EGFR, HER3, U3-1287, Cetuximab, Acquired cetuximab-resistance, Non-small cell lung cancer, MAPK, AKT

## Abstract

**Background:**

Cetuximab, an anti-EGFR monoclonal antibody, is used to treat several cancers. However, many patients who initially respond to cetuximab acquire resistance. To examine mechanisms of acquired resistance, we developed a series of cetuximab-resistant (Ctx^R^) clones derived from the cetuximab sensitive (Ctx^S^) non-small cell lung cancer (NSCLC) cell line H226. Previous studies characterizing this model revealed that: 1) EGFR was robustly overexpressed in Ctx^R^ clones due to decreased EGFR ubiquitination and degradation and 2) Ctx^R^ clones expressed increased HER2 and HER3 activation resulting in constitutive activation of the PI3K/AKT signaling axis. These findings suggest that dual targeting HER family receptors would be highly beneficial in the Ctx^R^ setting.

**Results:**

Since HER3 has been implicated in resistance to EGFR inhibitors, the efficacy of dually targeting both EGFR and HER3 in Ctx^R^ models was evaluated. First, EGFR and HER3 expression were knocked down with siRNAs. Compared to the Ctx^S^ parental cell line (HP), all Ctx^R^ clones exhibited robust decreases in cell proliferation upon dual knockdown. Analysis of Ctx^R^ clones indicated that neuregulin-1 was highly overexpressed compared to HP cells. Incubation of HP cells with neuregulin-1 rendered them resistant to cetuximab. Next, dual treatment of Ctx^R^ clones with cetuximab and the HER3 neutralizing monoclonal antibody (mAb) U3-1287 led to potent anti-proliferative effects. Blockade of EGFR with cetuximab resulted in inactivation of MAPK, while blockade of HER3 with U3-1287 resulted in the inactivation of AKT. Treatment with both mAbs resulted in knockdown of both signaling pathways simultaneously. HER2 was also strongly inactivated upon dual mAb therapy, suggesting that this treatment regimen can diminish signaling from three HER family receptors. *De novo* Ctx^R^ H226 mouse xenografts were established to determine if dual therapy could overcome acquired resistance to cetuximab in vivo. Tumors that had acquired resistance to cetuximab were significantly growth delayed upon dual treatment of U3-1287 and cetuximab compared to those continued on cetuximab only. Combinatorial-treated xenograft tumors expressed decreased Ki67 and increased cleaved caspase-3 levels compared to tumors treated with either monotherapy.

**Conclusions:**

These studies demonstrate that dually targeting HER family receptors with antibody-based therapies can overcome acquired resistance to cetuximab.

## Background

The HER family receptor tyrosine kinases (RTK) play critical roles in cell physiology, development, and cancer pathophysiology. This family consists of four members: EGFR, HER2, HER3 and HER4. These receptors are activated on the cell surface through binding to cognate ligands, leading to receptor homo- and hetero-dimerization with other HER family members. Dimerization of HER family receptors leads to the activation of each receptor’s tyrosine kinase, and subsequent activation of multiple downstream effector molecules [1, 2]. Specifically, EGFR regulates the RAS/RAF/MEK/ERK (also known as the MAPK) and PI3K/AKT signaling pathways, both of which have been attributed to increased cellular proliferation, survival, angiogenesis, and invasion.

Overexpression or hyperactivation of the EGFR is associated with poor prognosis in many human cancers, including metastatic colorectal cancer (mCRC), head and neck squamous cell carcinoma (HNSCC), non-small cell lung cancer (NSCLC) and brain cancer [3–6]. Therefore, targeting EGFR has been intensely pursued over the last three decades as a cancer treatment strategy. One approach uses monoclonal antibodies (mAbs) to target the extracellular domain of the EGFR to block natural ligand binding. Cetuximab (ICM-225, Erbitux) is a human: murine chimeric mAb that binds to extracellular domain III of the EGFR. This interaction partially blocks the ligand-binding domain and sterically hinders the correct extended conformation of the dimerization arm located on domain II [7]. The Food and Drug Administration (FDA) has approved cetuximab for treatment of patients with mCRC and HNSCC [8, 9], with more recent reports indicating clinical benefit for the treatment of NSCLC [10, 11]. Unfortunately, clinical data suggests that the majority of patients whom initially respond to cetuximab eventually acquire resistance [12–14].

Based on these clinical reports, a large effort has been undertaken to define the molecular mechanisms that underlie acquired resistance to cetuximab [15–22]. To do this, our laboratory has previously established a model of acquired resistance to cetuximab by exposing the cetuximab-sensitive (Ctx^S^) NSCLC cell line H226 to increasing concentrations of cetuximab until single cell resistant clones emerged [18]. Studies of this model cell system indicated that Ctx^R^ cells contained increased steady state expression and hyperactivation of the EGFR due to impaired internalization and degradation [18]. It was also shown that Ctx^R^ clones had increased EGFR-dependent activation of HER3 [18]. Consistent with this finding, several studies have revealed that resistance to anti-EGFR therapeutics may be due to subsequent activation of HER3 signaling pathways [23–26]. The HER3 receptor has been shown to be an important dimerization partner with EGFR, HER2 and c-MET which leads to sustained activity of the AKT signaling pathway [18, 23, 27–30]. Similar to EGFR, HER3 expression is associated with poor clinical outcome in lung, breast, ovarian and colon cancers [31–34]. These findings have led to the development of anti-HER3 therapeutics, one of which includes the fully humanized mAb U3-1287. Preclinical studies have indicated that U3-1287 binding to the extracellular region of HER3 led to its internalization and subsequent degradation in models of breast, lung, and head and neck cancer [35–37].

In the current study, we hypothesized that Ctx^R^ clones and tumors may benefit by the dual targeting of EGFR and HER3. We found that cetuximab and U3-1287 dual therapy led to the inhibition of cellular proliferation and survival pathways in Ctx^R^ clones, while single agent therapy had minimal effect. Caspase-3/7 and annexin-V assays indicated a significant increase in apoptosis in cells treated with dual therapy compared to either agent alone. Moreover, growth of Ctx^R^ tumors treated with cetuximab and U3-1287 were significantly delayed compared to mice continued on cetuximab monotherapy. Analysis of combinatorial-treated xenograft tumors demonstrated decreased activation of HER2, HER3 and Ki67 as well as a modest increase in cleaved caspase-3 compared to tumors treated with monotherapy. The results presented herein suggest that dual targeting HER family receptors with antibody-based therapies can overcome acquired resistance to cetuximab.

## Results

### Cetuximab-resistant (Ctx^R^) clones have increased HER family receptor activity

**Figure 1:**
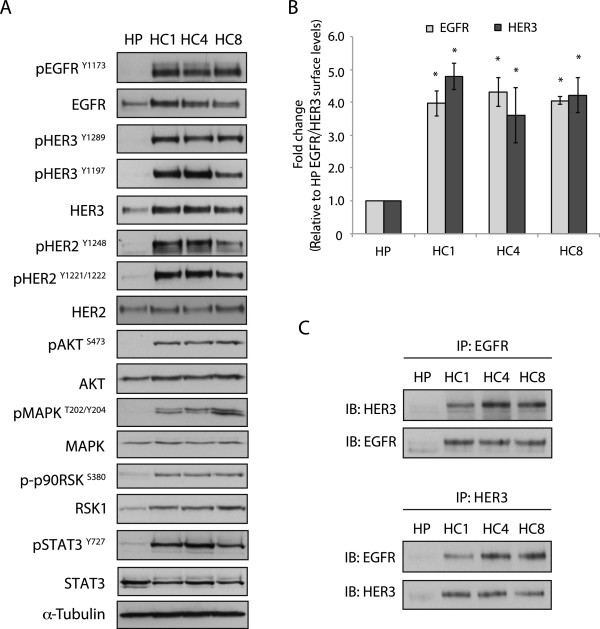
**Cetuximab-resistant (Ctx** ^**R**^ **) clones have increased HER family receptor activity. (A)** Ctx^R^ cells have increased HER family activity as well as phosphorylation levels of downstream signaling molecules. Parental (HP) and Ctx^R^ clones (HC1, HC4, HC8) were harvested and protein lysates were fractionated on SDS-PAGE followed by Immunoblotting for the indicated proteins. α-Tubulin was used as a loading control. **(B)** Expression of EGFR and HER3 on the plasma membrane is increased in Ctx^R^ clones compared to cetuximab-sensitive (Ctx^S^) cells (HP). The levels of EGFR and HER3 on the cell surface were measured by flow cytometry. (n = 3) **(C)** EGFR displayed increased association with HER3 in all three Ctx^R^ clones compared to Ctx^S^ HP cell line. Cells were harvested and EGFR or HER3 were immunoprecipitated with anti-rabbit EGFR antibody or anti-rabbit HER3 antibody. The immunoprecipitate complexes were fractionated on SDS-PAGE followed by immunoblotting for indicated proteins.

We previously reported that Ctx^R^ clones (HC1, HC4 and HC8) exhibited increased expression and activity of EGFR relative to the Ctx^S^ parental control cells (HP) [18]. In addition, HER3 expression and phosphorylation were increased in Ctx^R^ clones, as well as the phosphorylation of HER2 and the downstream effector molecules AKT, MAPK, p90-RSK and STAT3 (Figure 1A). Quantifying EGFR and HER3 on the cell surface by flow cytometry indicated that there was greater plasma membrane expression of EGFR and HER3 in Ctx^R^ clones compared to HP cells (Figure 1B). Further, immunoprecipitation analysis indicated that EGFR and HER3 were highly associated in Ctx^R^ clones, while this association was absent in Ctx^S^ HP cells (Figure 1C). Taken together these data suggest that prolonged exposure to cetuximab leads to increased expression and association of EGFR and HER3, as well as increased activation of HER2.

### Ctx^R^ clones depend on EGFR and HER3 for proliferation and cetuximab resistance

**Figure 2:**
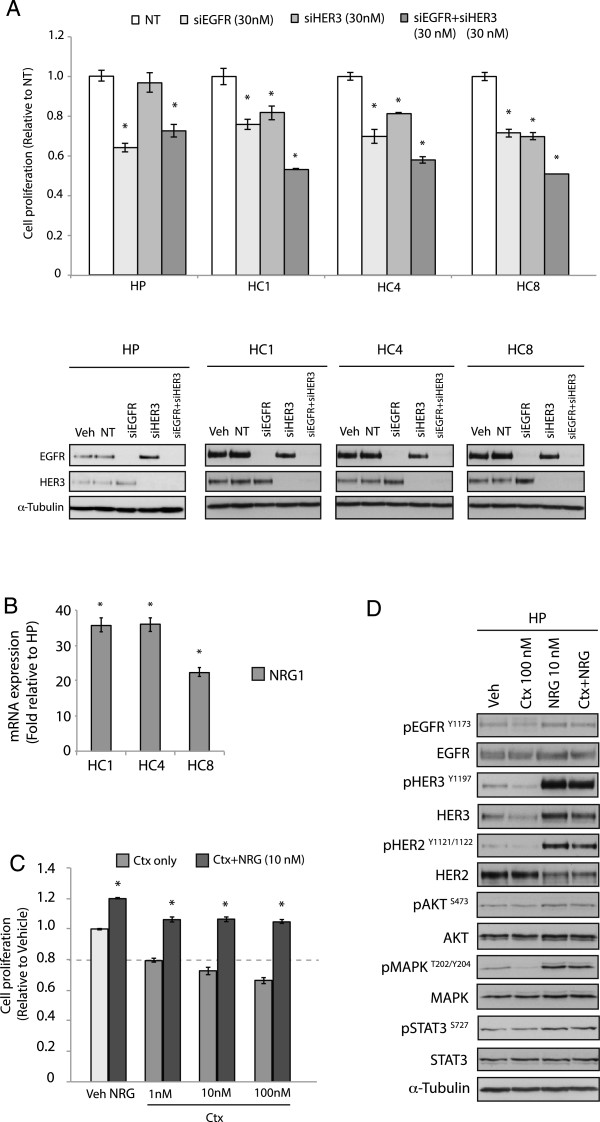
**Ctx** ^**R**^ **clones depend on EGFR and HER3 for proliferation and cetuximab response. (A)** Effects of EGFR and HER3 knockdown on proliferation of Ctx^R^ cells. Proliferation was measured at 72 h after treatment using the crystal violet assay. Data points are represented as mean ± s.e.m (n = 3). *p ≤0.05. Whole cell lysates were harvested after 72 h treatment and fractionated on SDS-PAGE followed by immunoblotting for the indicated proteins. α-Tubulin was used as a loading control. **(B)** Upregulation of NRG-1 ligand in Ctx^R^ clones by real-time qPCR. NRG-1 expression level in Ctx^R^ clones HC1, HC4 and HC8 was measured by real-time qPCR analysis. Data are represented as fold increase relative to the HP parental control. Data points are represented as mean ± s.e.m. (n = 4). **(C)** NRG-1 ligands can enhance cetuximab resistance in cetuximab-sensitive cells. Proliferation was measured at 72 h after treatment using CCK8 assays and plotted as a percentage of proliferation relative to the vehicle cells. Data points are represented as mean ± s.e.m. (n = 4). **(D)** NRG-1 increased phosphorylation levels of HER family receptors and their respective kinase targets in HP cells. Whole cell lysates were harvested and fractionated on SDS-PAGE followed by immunoblotting for the indicated proteins. α-Tubulin was used as a loading control.

To determine if cells with acquired resistance to cetuximab depend on EGFR and/or HER3 signaling, proliferation assays using small interfering RNAs (siRNA) targeting EGFR and HER3 (Figure 2A) were performed. All three Ctx^R^ clones showed greater inhibition of proliferation when transfected with both siRNAs compared to either siRNA alone. Interestingly, siHER3 alone did not augment proliferation, indicating minimal utility of targeting only HER3 in Ctx^R^ clones. Immunoblot analysis validated knockdown of each target per treatment group (Figure 2A).

Since siHER3 impaired the proliferation of Ctx^R^ clones only upon knockdown of EGFR expression, we next sought to identify if ligand induced activation of HER3 may mediate cetuximab resistance. First, quantitative PCR (qPCR) was used to analyze neuregulin-1 (NRG-1) expression in Ctx^R^ clones compared to HP parental cells (Figure 2B), where there was a 20–40 fold increase in NRG-1 expression in all Ctx^R^ clones. This large increase in HER3 ligand expression suggested that autocrine activation of HER3 may mediate cetuximab resistance. To test this hypothesis, we next stimulated the Ctx^S^ parental HP cells with NRG-1 to assess if this would lead to increased resistance to cetuximab. Addition of NRG-1 to HP cells resulted in resistance to increasing doses of cetuximab treatment (Figure 2C). Analysis of downstream signaling molecules indicated that HP cells stimulated with NRG-1 had robust activation of all three HER family receptors, in addition to AKT and MAPK effector kinases. The activation of these molecules was not inhibited by cetuximab in the presence of NRG-1. This result suggests that activation of HER3 can stimulate cells to regulate cell proliferation and survival pathways, and thus bypass the inhibitory effects of cetuximab. Collectively, these data indicate that HER3 cooperates with EGFR to regulate cellular proliferation and cetuximab response.

### Dual blockade of HER3 and EGFR can effectively inhibit the proliferation of Ctx^R^ clones

**Figure 3:**
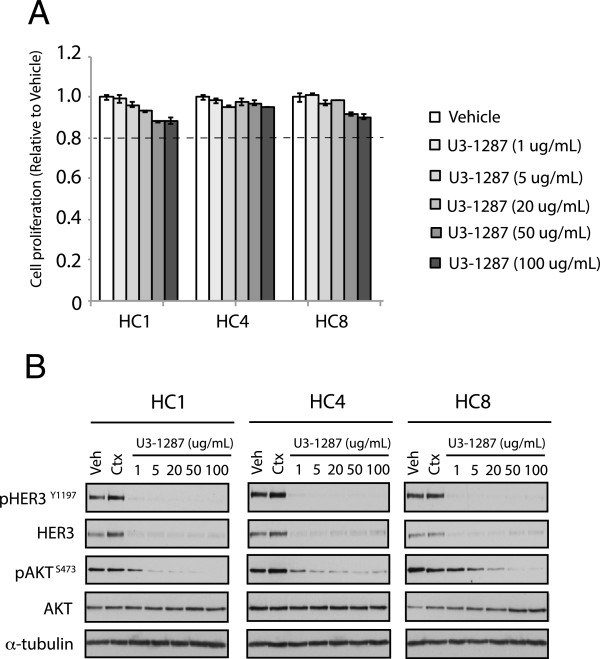
**U3-1287 downregulates total and phosphorylation of HER3 as well as AKT phosphorylation, but does not inhibit cell proliferation in Ctx** ^**R**^ **clones. (A)** U3-1287 alone did not inhibit cell proliferation in Ctx^R^ clones. The cell proliferation was measured via crystal violet assay and plotted as a percentage of proliferation relative to the vehicle control cells. Data points are represented as mean ± s.e.m. (n = 3). **(B)** U3-1287 downregulates total HER3 and phosphorylation of AKT in Ctx^R^ clones. Whole cell lysates were fractionated on SDS-PAGE followed by immunoblotting for the indicated proteins. α-Tubulin was used as a loading control.

To further investigate if HER3 plays a role in Ctx^R^ cellular proliferation, Ctx^R^ clones were treated with increasing concentrations of the anti-HER3 monoclonal antibody U3-1287 for 72 h. Results of this experimentation indicated that U3-1287 monotherapy did not significantly affect cellular proliferation of Ctx^R^ clones (Figure 3A), consistent with siHER3 data presented in Figure 2A. Examination of U3-1287 treated cells indicated that HER3 was effectively degraded upon treatment, even at low doses (1 ug/mL). A dose dependent decrease in the phosphorylation of AKT on S473 was also observed. Overall, these findings indicate that U3-1287 can effectively target HER3 in Ctx^R^ clones however, there was minimal effect on proliferation by monotherapy.

**Figure 4:**
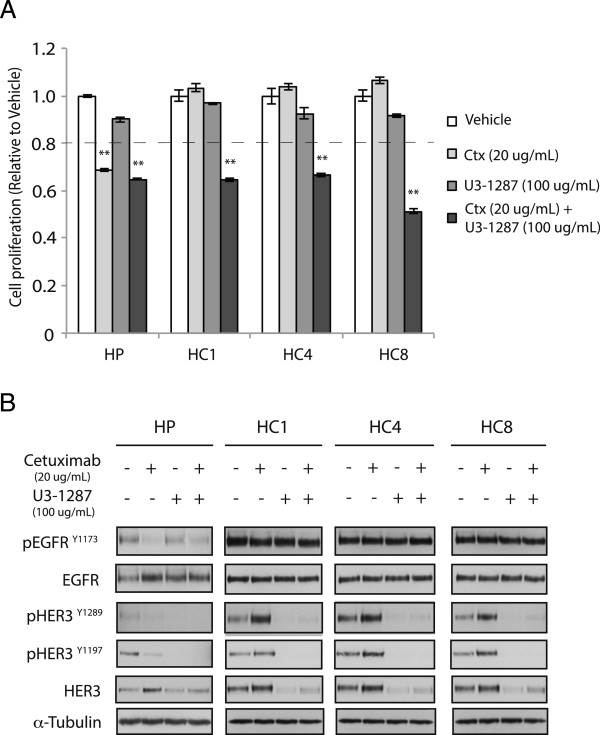
**Dual blockade of HER3 and EGFR can effectively inhibit the proliferation of Ctx** ^**R**^ **clones. (A)** Combinatorial treatment of Ctx^R^ clones with cetuximab and U3-1287 leads to proliferation inhibition. Cell proliferation was measured using crystal violet assay and plotted as a percentage of proliferation relative to the vehicle control cells. Data points are represented as mean ± s.e.m. (n = 3). **(B)** Combinatorial treatment with cetuximab and U3-1287 leads to loss of HER3 expression in Ctx^R^ clones. Protein lysates were fractionated on SDS–PAGE followed by immunoblotting for the indicated proteins. α-Tubulin was used as a loading control.

Since dual knockdown of EGFR and HER3 led to potent anti-proliferative effects in Ctx^R^ clones (Figure 2A), we hypothesized that cetuximab in combination with U3-1287 may produce a anti-proliferative response. Thus, cell proliferation analysis was performed after treatment of Ctx^R^ clones with vehicle, cetuximab (20 ug/ml), U3-1287 (100 ug/mL) or the combination of cetuximab and U3-1287. The results of these experiments indicated that while cetuximab or U3-1287 monotherapy did not affect the proliferation of Ctx^R^ clones, the combination of the two drugs showed significant anti-proliferative effects (Figure 4A).

We further investigated if EGFR or HER3 activation was inhibited by combined treatment with U3-1287 and cetuximab. Ctx^R^ clones were treated with vehicle, 20 ug/mL cetuximab, 100 ug/mL U3-1287 or combinatorial treatment for 24 h. Analysis of EGFR and HER3 protein levels post treatment indicated that HER3 was robustly degraded by U3-1287 or dual treatment in Ctx^R^ clones, while total and activated EGFR (Y1173) was not affected with either treatment (Figure 4B). Interestingly, treatment with cetuximab led to modest increases in expression of total levels of HER3 in both Ctx^R^ clones and HP cells as well as phopho-HER3 levels in Ctx^R^ clones.

### Combined treatment with cetuximab and U3-1287 leads to impaired HER2, AKT and MAPK signaling

**Figure 5:**
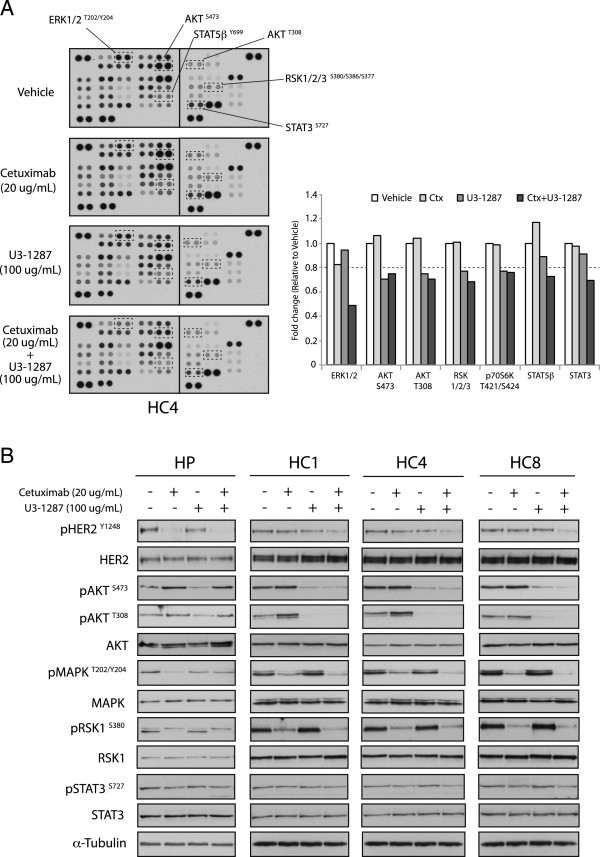
**Combined treatment of Ctx** ^**R**^ **clones with cetuximab and U3-1287 inhibits HER2, AKT and MAPK signalings more effectively than either drug alone. (A)** Human Phospho-Kinase array analysis demonstrated that combined treatment with cetuximab and U3-1287 inhibits proliferation and survival signaling in Ctx^R^ cell clone, HC4. The cell extracts were incubated with membranes containing antibodies to 46 different kinase phosphorylation sites. Quantitation of phosphorylated proteins was completed using scanned images from ImageJ software. Data points are represented as the mean of duplicate spots. **(B)** Effects of combined cetuximab and U3-1287 treatment on their respective kinase targets in Ctx^R^ clones. Protein lysates from Figure 5A (HC4) were fractionated on SDS–PAGE followed by immunoblotting for the indicated proteins. Protein lysate from other Ctx^R^ clones (HC1 and HC8) as well as HP cells were obtained after treatment with vehicle, cetuximab (20 ug/mL), U3-1287 (100 ug/mL) or the combination of cetuximab and U3-1287 for 24 h. α-Tubulin was used as a loading control.

Since the combination of U3-1287 and cetuximab resulted in strong anti-proliferative effects in Ctx^R^ clones (Figure 4A), we investigated which downstream pathways were impaired. Human Phospho-Kinase array analysis on Ctx^R^ clone (HC4) treated with vehicle, cetuximab (20 ug/mL), U3-1287 (100 ug/mL) or the combination of cetuximab and U3-1287 for 24 hours was performed. This human Phospho-Kinase array contains antibodies to 46 different phosphorylated kinases involved in cellular proliferation and survival. Quantitation of phosphorylated proteins was completed using scanned images from ImageJ software for each treatment and were summarized in Figure 5A. Combined treatment with U3-1287 and cetuximab inhibited several downstream EGFR and HER3 pathways including pERK1/2 (pMAPK), pAKT, pRSK1/2/3, STAT5β, and STAT3. The inhibition of these downstream targets was validated in all three Ctx^R^ clones and HP cells by immunoblot analysis. Combinatorial treatment of U3-1287 and cetuximab for 24 hours resulted in decreased levels of phosphorylated AKT, MAPK, RSK1 and STAT3 in Ctx^R^ clones (Figure 5B). Phosphorylation of HER2 on tyrosine 1248 (Y1248) in HP cells was inhibited by cetuximab treatment alone, while HER2 phosphorylation in all three Ctx^R^ clones was strongly inhibited only by the combination treatment. These results indicate that the dual targeting of EGFR and HER3 lead to a robust inhibition of HER2 as well as AKT and MAPK signaling pathways.

### Combined treatment with cetuximab and U3-1287 promotes apoptosis in Ctx^R^ clones

**Figure 6:**
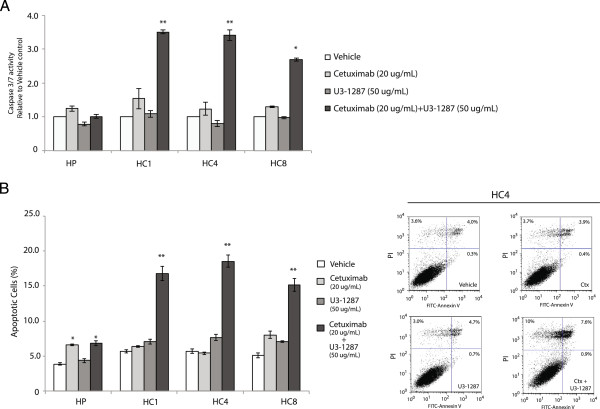
**Cetuximab and U3-1287 induced apoptosis in Ctx** ^**R**^ **clones. (A)** Combinatorial treatment with cetuximab and U3-1287 activates Caspase 3/7 compared to either drug alone in Ctx^R^ clones. Caspase-3/7 activity was determined by Caspase 3/7-Glo assay. Data represent means ± s.e.m from 3 independent experiments (n = 9). *p <0.05 or **p <0.01. **(B)** The percentage of Annexin v-positive/propidium iodide-negative cells was increased significantly after the combination of cetuximab and U3-1287 treatment compared to vehicle, cetuximab or U3-1287 alone in the Ctx^R^ clones. Cells were plated and allowed to adhere for 24 h prior to treatment with vehicle, cetuximab (20 ug/mL), U3-1287 (50 ug/mL) or the combination of cetuximab (20 ug/mL) and U3-1287 (50 ug/mL) for 24 h prior to Annexin-v analysis via flow cytometry. Data points are represented as mean ± s.e.m. (n = 3). *p <0.05 or **p ≤0.001. Flow cytometry profile in HC4 cells represents Annexin-V-FITC staining in x-axis and PI in y-axis. The number represents the percentage of cells in each condition.

We demonstrated that combined EGFR and HER3 knockdown inhibited cell proliferation in Ctx^R^ clones more than knockdown of either gene alone. To see if combinatorial treatment of Ctx^R^ clones led to increased apoptosis, caspase-3/7 activity and Annexin-V analyses were conducted after 24 h treatment with vehicle, 20 ug/mL cetuximab, 50 ug/mL U3-1287 or both antibodies. Ctx^R^ clones treated with dual therapy demonstrated robust increases in caspase-3/7 activity (~2-3 fold) indicative of cells actively undergoing apoptosis, while single therapy treatment did not increase caspase-3/7 activity over vehicle treated cells (Figure 6A). Further, Annexin-V analysis by flow cytometry indicated statistically significant increases in apoptosis by combination treatment in all Ctx^R^ clones (16-19%) compared to single therapy or vehicle treatment (Figure 6B). Collectively, these data indicate that combination treatment of Ctx^R^ clones with U3-1287 and cetuximab can induce caspase-dependent apoptosis.

### The combination treatment of cetuximab and U3-1287 can overcome acquired resistance to cetuximab in vivo

**Figure 7:**
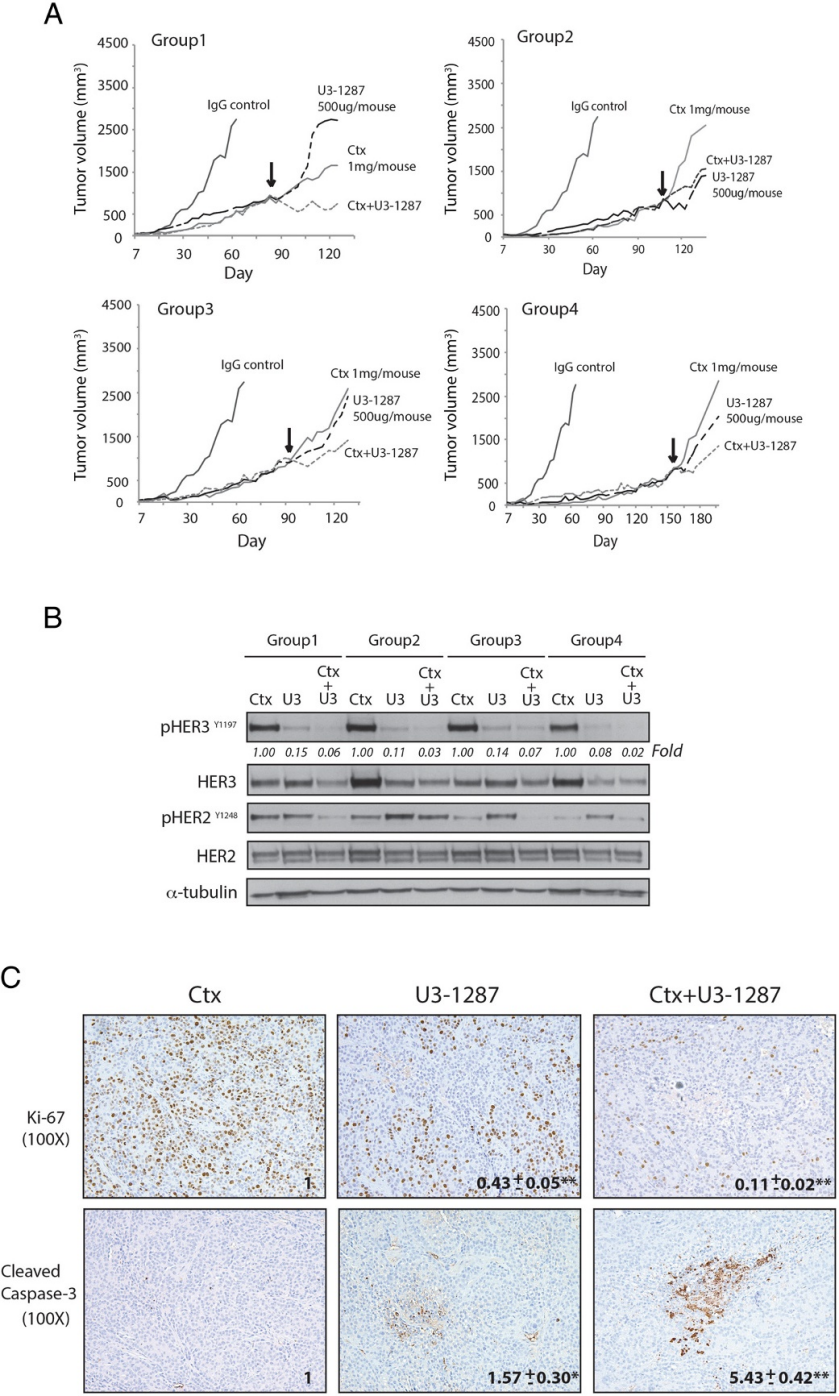
**Combination of cetuximab and U3-1287 treatment of Ctx** ^**R**^ **tumors leads to growth delay in vivo. (A)** Growth-delay effects of U3-1287 in Ctx^R^ tumors in vivo. The black arrow designates the starting time point of U3-1287 treatment. The average tumor volume of mice treated with IgG is included in all groups for comparison purposes. **(B)** Combination treatment with cetuximab and U3-1287 inhibited HER3 expression and HER2 activation in vivo. Total and phosphorylation levels of HER2 and HER3 proteins in Ctx^R^ xenograft tumors were determined by immunoblot analysis after cetuximab, U3-1287 or the combination of cetuximab and U3-1287 treatments. **(C)** The inhibition of phospho-HER3 and phospho-HER2 expression in Ctx^R^ tumors after combinatorial treatment corresponds with reduced proliferation and increased apoptosis. Ctx^R^ tumor samples after cetuximab, U3-1287 or the combination of cetuximab and U3-1287 treatment in vivo were prepared and analyzed for Ki67 and cleaved caspase 3 by immunohistochemistry. Images were quantified via taking the average staining intensity measured from 3 tumors per treatment group (3 images/tumor, n = 9). Magnification 100X.

To evaluate the efficacy of U3-1287 and cetuximab combination treatment against tumor growth in vivo, a series of mouse xenograft studies of *de novo* acquired resistance to cetuximab [15, 38] were established. To develop acquired resistance to cetuximab in vivo, we inoculated 40 mice with the NSCLC line H226 unilaterally with 2 × 10^6^ cells in the dorsal flank. Tumors were allowed to grow to 100 mm^3^, at which time 30 mice were treated with cetuximab (1 mg/mouse) twice weekly and 10 mice were treated with IgG control (1 mg/mouse) twice weekly by intraperitoneal injection. IgG treated tumors grew uninhibited, while cetuximab treated tumors demonstrated tumor control and delayed growth. Tumors were monitored for the development of cetuximab resistance, defined as marked tumor growth in the presence of continued cetuximab therapy. Once Ctx^R^ tumors reached a volume of ~800 mm^3^, mice were grouped according to tumor size at the time of resistance. Ctx^R^ was observed in 20 of 30 tumor xenografts (67%) treated with cetuximab, similar to previous studies from our laboratory [15, 38]. Thus, a total of six Ctx^R^ mouse xenograft groups were selected for further study (18 mice in total). Upon establishment of Ctx^R^ mouse groups, one mouse was maintained on cetuximab (1 mg), one mouse was removed from cetuximab and started on U3-1287 (500 ug) mono-therapy, and another mouse was given the combination treatment. The average tumor volume of mice treated with IgG alone is included in all groups for comparison purposes. Four out of 6 (67%) Ctx^R^ tumors treated with U3-1287 and cetuximab demonstrated a tumor growth delay compared to the mice that were maintained on cetuximab monotherapy, while 2 (33%) tumors failed to respond to U3-1287. In Figure 7A, the black arrow designates the starting time point of U3-1287 treatment. Mice treated with cetuximab and U3-1287 in Groups 1, 3, and 4 demonstrated more robust anti-proliferative response than tumors maintained on cetuximab or switched to U3-1287 monotherapy. This anti-tumor response was maintained for more than 30 days in the dually treated mice. In contrast the tumor treated with U3-1287 and cetuximab in Group 2 did not exhibit delayed tumor growth compared to the tumor treated with U3-1287 alone. Analysis of tumor lysates harvested from each treatment group indicated that phosphorylated HER3 was significantly reduced in all tumors from U3-1287 treated mice, while mice treated with dual therapy exhibited even greater reductions in both total and phosphorylated HER3 levels (Figure 7B). Additionally, the mice treated with dual therapy that demonstrated anti-proliferative responses in Figure 7A also expressed less phosphorylated HER2 (Figure 7B). This observation may explain why U3-1287 and cetuximab dual combination was more potent in these mice. Next, the proliferation and apoptotic index of tumors from each treatment group were examined by immunohistochemistry (Figure 7C). Ki67, a marker of actively proliferating cells, was robustly reduced in tumors treated with dual therapy, while cleaved caspase 3, a marker of cells actively undergoing apoptosis, was significantly increased in these tumors. Together, these data demonstrate that *de novo* Ctx^R^ tumor xenografts can be sensitized to cetuximab induced growth delay upon inhibition of HER3 activity with U3-1287.

## Discussion

While many patients initially respond to the anti-EGFR mAb cetuximab, substantial clinical gains have not been observed because resistance is inevitably the outcome. In an attempt to study mechanisms of resistance, several model systems have been established where cetuximab sensitive cells are treated with increasing doses of cetuximab until resistant clones emerge. In the current study, we found that EGFR and HER3 cooperate in cetuximab resistant clones derived from the NSCLC cell line H226. Targeting both HER3 and EGFR with the neutralizing anti-HER3 mAb U3-1287 in combination with cetuximab led to robust anti-proliferative effects and modest apoptotic effects in Ctx^R^ clones in vitro and in *de novo* models of acquired resistance to cetuximab in vivo. These data suggest that dual targeting HER family receptors with antibody-based therapies can overcome acquired resistance to cetuximab and provides rationale for the clinical movement of this therapeutic strategy for the treatment of cetuximab-resistant cancers.

HER3 is now known to play a major role in resistance to both anti-HER2 and anti-EGFR therapeutics. In addition to the current model, several investigators have observed compensatory increases in HER3 activation post targeting alternative HER receptors, indicating that HER3 can maintain signaling pathways necessary for cancer cell proliferation and survival when other HER receptors are inhibited [24, 39, 40]. In the current study we found that dual targeting both HER3 and EGFR led to a significant loss in cellular proliferation (Figure 4A) and delayed growth of Ctx^R^ tumors (Figure 7A). Interestingly, Ctx^R^ tumors in Group 2 were sensitive to U3-1287, but U3-1287 addition to cetuximab did not provide any further benefit (Figure 7A). Analysis of tumor lysate harvested from U3-1287 and cetuximab treated mice in Group 2 indicated that HER3 activation and expression was effectively downregulated, however, HER2 activity was not (Figure 7B). The lack of HER2 blockade may be a reason why no further benefit was detected upon dual therapy in Group 2. While the current study focused on targeting the HER3 receptor as a method to overcome cetuximab resistance, previous studies indicate that targeting HER2 can also sensitize Ctx^R^ clones to cetuximab, with a parallel loss in HER3 activity [18]. These findings highlight the necessity of targeting both EGFR and the HER2:HER3 signaling axes to achieve maximal anti-tumor responses. Our findings are corroborated by independent studies in HNSCC, CRC, and gastric cancer, where dual targeting of HER family receptors can effectively overcome resistance to anti-HER family monotherapies [41–43]. Collectively, our data indicate that targeting multiple HER receptors is necessary for the complete inhibition of the HER family signaling network, and may thus be a beneficial treatment strategy for patients that have acquired resistance to cetuximab.

The importance of knock down multiple HER family receptors is highlighted by analyzing oncogenic signaling pathways abrogated post dual receptor inhibition. In the current study, cetuximab treatment of Ctx^R^ clones resulted in reduced MAPK activity, while U3-1287 resulted in reduced AKT activity (Figure 5B); the combination of both mAbs led to the simultaneous knockdown of both pathways. The importance of inhibiting both signaling pathways is highlighted by the fact that single HER family mAb therapy did not augment Ctx^R^ cell proliferation or survival (Figures 4 and 6). We speculate that Ctx^R^ cells have compensatory mechanisms that allow for sustained proliferation and survival upon knockdown of either the AKT or MAPK signaling pathways, highlighting the importance of targeting both pathways simultaneously. Additionally, there was a significant increase in apoptosis post dual therapy in Ctx^R^ clones but not in Ctx^S^ parental cells (Figure 6). This finding suggests that Ctx^R^ cells are more highly dependent on both cell survival and proliferation pathways, supporting the necessity of dual targeting both AKT and MAPK. Interestingly, HER2 activity was abrogated by dual therapy in Ctx^R^ cells (Figure 5B), further demonstrating the importance of dual targeting HER family receptors to maximally inhibit the HER family signaling network.

Neuregulin-1 (NRG-1) is the predominant ligand responsible for binding and activating HER3. Previous reports from our laboratory have identified that EGFR ligands, including EGF, heparin binding EGF, amphiregulin, and β-cellulin were upregulated 2–9 fold in Ctx^R^ clones [16]. In the current study, we found that Ctx^R^ clones expressed 20–40 fold more NRG-1 compared to the Ctx^S^ parental cells by qPCR (Figure 2B), where the addition of NRG-1 to the Ctx^S^ parental cell line rendered these cells resistant to cetuximab (Figure 2C). These data indicate that NRG-1 autocrine signaling is a major driver of cetuximab resistance in this model, which was subsequently prevented through the degradation of HER3 upon treatment with U3-1287. Similar to our findings, increased NRG-1 secretion has been attributed to gefitinib resistant breast cancer cells [44], and recently NRG-1 rendered cells insensitive to trastuzumab-DM1 [45]. Furthermore, several growth factors have been shown to enhance resistance to a plethora of different tyrosine kinase inhibitors in cell line models [46]. Interestingly, increased HER family ligands were shown to mediate resistance to anti-AKT inhibitors in triple-negative breast cancer cells, which was abrogated by targeting both EGFR and HER3 with a dual receptor ligand-blocking antibody [47]. In attempt to combat increased HER family ligand expression and autocrine activation of HER receptors, mAbs targeting HER family ligands may serve as potential therapeutics by sequestering ligands prior to their activation of HER receptors. One research group has developed a novel bispecific mAb that can sequester EGFR ligands and NRG-1, resulting in anti-proliferative effects in several cancer models [48]. Recently, a novel study generated mAbs directed against NRG1 that demonstrated potent anti-tumor effects in combination with chemotherapy in NSCLC models, and prevented the expansion of residual tumor cells post chemotherapy regimes [49]. Since excess HER family ligands can manifest in cetuximab resistance (Figure 2C and [16]), the use of mAb’s targeting HER family ligands in combination with anti-HER family mAb’s may be highly beneficial in the Ctx^R^ setting. Overall, autocrine stimulation of HER receptors may play a critical role in resistance to numerous therapeutic modalities, and strengthens the need for dual targeting HER family receptors.

Currently, there are several anti-HER3 mAbs being evaluated for the treatment of cancer patients. Recent studies investigating the use of a bispecific antibody against EGFR and HER3, MEHD7945A, demonstrated robust capability of overcoming resistance to both erlotinib and cetuximab in NSCLC and HNSCC cell models [50]. While MEHD7945A was capable of reducing the activation of HER3 in these cell systems, total HER3 levels remained constant. Neutralizing mAbs, such as U3-1287, may have greater potential to elicit more robust anti-tumor effects in a variety of cancers due to their ability to elicit receptor internalization and degradation rather than inhibiting ligand-induced activity. The effective degradation of a receptor will bypass resistant mechanisms such as receptor mutation, ligand overabundance, and ligand-independent oncogenic properties of RTKs. These mechanisms of resistance have borne out in several studies with mAbs that only inhibit the ligand-induced activity of EGFR such as the recent identification of EGFR ligand binding domain mutations in cetuximab resistant tumors [51]. Lantto et al. also demonstrated that Sym013, a mixture of monoclonal antibodies targeting EGFR, HER2 and HER3, inhibited proliferation in a large number of cancer cell lines in vitro as well as multiple xenograft models [52]. Sym013 resulted in the effective degradation of all three HER family receptors, and prevented the compensatory upregulation of alternative RTKs both in vitro and in vivo modeling. Overall, cocktails of neutralizing mAbs that elicit total HER family receptor degradation may overtake the future of the HER family targeting field.

Pre-clinical data suggests strong anti-tumor responses in several cancer settings with U3-1287 in combination with trastuzumab, and most recently in combination with radiation therapy [53, 54]. Furthermore, U3-1287 is the most clinically advanced anti-HER3 mAb, where it has been deemed safe and tolerable in phase I/II clinical trials in advanced solid tumors [55, 56]. U3-1287 is currently undergoing phase II trials in combination with trastuzumab and paclitaxel in metastatic breast cancer, and has now progressed to phase III clinical trials in NSCLC (clinicaltrials.gov). Cumulatively, our data as well as others, indicate that dual targeting HER family receptors may induce robust anti-tumor responses, and thus may be a beneficial treatment strategy for cetuximab resistant cancers.

## Conclusions

In the current study, we found that EGFR and HER3 collaborate in cetuximab resistant clones derived from the NSCLC cell line H226. Targeting both HER3 and EGFR with the neutralizing anti-HER3 monoclonal antibody U3-1287 in combination with cetuximab led to robust anti-proliferation effects and modest apoptotic effects in Ctx^R^ clones in vitro and in *de novo* models of acquired resistance to cetuximab in vivo. Our data suggest that dual targeting HER family receptors with antibody-based therapies can overcome acquired resistance to cetuximab and provide rationale for the clinical movement of this therapeutic strategy for the treatment of cetuximab-resistant cancers.

## Materials and Methods

### Cell lines

The human NSCLC cell line H226 was provided by Drs. Minna J. and Gazdar A. (University of Texas Southwestern Medical School, Dallas, TX). The cells were maintained in 10% fetal bovine serum in RPMI-1640 (Mediatech Inc., Manassas, VA, USA) with 1% penicillin and streptomycin. The development of Ctx^R^ clones has been previously described [18].

### Small interfering RNA and transfection

For small interfering RNAs (siRNAs), Ctx^R^ cells (HC1, HC4 and HC8) were transiently transfected with siEGFR (ON-TARGETplus, SMART pool #L-003114-00, Dharmacon, Lafayette, CO, USA) and siHER3 (ON-TARGETplus, SMART pool #L-003127-00, Dharmacon) using Lipofectamine RNAiMAX according to the manufacturer's instructions (Invitrogen, Carlsbad, CA, USA). The non-targeting siRNA (ON-TARGETplus Non-targeting Pool, #D-001810-10) was obtained from Dharmacon as a control. Cells were then lysed for analysis of protein knockdown by immunoblotting after siRNA transfection.

### Materials

Neuregulin 1 was obtained from R&D Systems (Minneapolis, MN, USA). Cetuximab (ICM-225, Erbitux) was purchased from University of Wisconsin Pharmacy. U3-1287 was generously provided by U3 Pharma GmbH (Martinsried, Germany).

### Antibodies

All antibodies were purchased from commercial sources as indicated below: EGFR, pEGFR (Y1173), HER2, HER3 and HRP-conjugated goat-anti-rabbit IgG and goat-anti-mouse IgG were obtained from Santa Cruz Biotechnology Inc. (Dallas, TX, USA). pHER3 (Y1289), pHER3 (Y1197), pHER2 (Y1248), pAKT (S473, T308), pMAPK (T202/Y204), MAPK, p-p90RSK (S380), RSK1/2/3, pSTAT3 (S727), STAT3, cleaved caspase-3 and Ki67 were obtained from Cell Signaling Technology (Danvers, MA, USA). α-Tubulin was purchased from Calbiochem (Billerica, MA, USA).

### Cell proliferation assay

Equal numbers of cells were seeded in 6 well plates. Following treatment, monolayers were washed with PBS and fixed/stained with 0.5% crystal violet. Plates were air dried overnight and dye was eluted with 0.1 M sodium citrate (pH 4.2) in ethanol (1:1). Elution was transferred to 96-well plates, and the absorbance was read at 540 nm to determine cell proliferation. All treatments were performed in triplicate. Cells were also seeded at 2,000 cells per well in 100 μl of media on a 96 well plate, grown for 24 h and then treated with drug for 72 h prior to analysis using the Cell Counting Kit 8 (Dojindo Molecular Technologies, Rockville, MD) according to the manufacture’s instructions. All treatments were performed in quadruplicate.

### Immunoblotting analysis

Whole cell protein lysate was obtained by tween-20 lysis buffer (50 mM HEPES, pH 7.4, 150 mM NaCl, 0.1% Tween-20, 10% glycerol, 2.5 mM EGTA, 1 mM EDTA, 1 mM DTT, 1 mM Na_3_VO_4_, 1 mM PMSF, 1 mM BGP and 10 μg/ml of leupeptin and aprotinin). Samples were sonicated and then centrifuged at 15,000 g for 10 min at 4°C. Protein concentrations were determined by Bradford assay (Bio-Rad Laboratories, Hercules, CA, USA). Equal amounts of protein were fractionated by SDS-PAGE, transferred to a PVDF membrane (Millipore, Billerica, MA, USA), and analyzed by incubation with the appropriate primary antibody. Proteins were detected via incubation with HRP-conjugated secondary antibodies and ECL Western Blotting Substrate (Promega Cooperation, Madison, WI, USA), SuperSignal* West Dura Extended Duration Chemiluminescent Substrate or SuperSignal* West Femto Maximum Sensitivity Chemiluminescent Substrate (Thermo Fisher Scientific, Waltham, MA, USA).

### Immunoprecipitation

Cells were lysed with NP-40 lysis buffer (50 mM HEPES, pH 7.4, 150 mM NaCl, 1% NP-40, 0.5% deoxycholic acid, 10% glycerol, 2.5 mM EGTA, 1 mM EDTA, 1 mM DTT, 1 mM PMSF, 1 mM BGP and 10 mg/ml of leupeptin and aprotinin). Cell lysates containing 0.5 mg of protein were incubated overnight at 4°C with 200 ug/mL of anti-rabbit EGFR or anti-rabbit HER3 antibodies. After adding 30 uL of protein A/G agarose beads (Santa Cruz), cell lysates were incubated for another 2 h at 4°C. The immunoprecipitates were pelleted by centrifugation and washed several times with NP-40 lysis buffer. The captured immunocomplexes were then eluted by boiling the beads in 2xSDS sample buffer for 5 min and subjected to immunoblot analysis as described above.

### cDNA synthesis and qPCR

Total RNA from cells was prepared using an RNeasy Mini kit (Qiagen, Inc., Valencia, CA). cDNA from total RNA of HP, HC1, HC4 and HC8 were synthesized using qScript cDNA SuperMix (Quanta Biosciences, Gaithersburg, MD, USA). qPCR analysis was performed using a Bio-Rad CFX96 Real-Time PCR Detection System (Bio-Rad Laboratories) using the iQ Supermix as recommended by manufacturer. All reactions were performed in quadruplicate. The NRG1 primer sets (Hs00247624_m1) used for this analysis were purchased from Life Technologies TaqMan Gene Expression Assay. Fold increases in gene expression were determined by quantitation of cDNA from target samples (HC1, HC4 and HC8) relative to a calibrator sample (HP). Human β-actin gene (F: 5′-CAGCCATGTACGTTGCTATCCAGG-3′, R: 5′-AGGTCCAGACGCAGGATGGCATG-3′) was used as the endogenous control for normalization of initial RNA levels. To determine this normalized value, 2^-ΔΔCT^ values were compared references between target and calibrator samples, where the change in crossing threshold ΔCt = Ct_NRG1_-Ct_β-actin_ and ΔΔCt = ΔCt_HC1, HC4 or HC8_-ΔCt_HP_.

### Flow cytometric analysis

Cells were washed in PBS and harvested with PBS-EDTA (0.02% solution). A cell suspension containing 1 × 10^6^ cells in 100 uL flow buffer (PBS containing 1% FBS) was incubated with 1 ug of either control IgG, anti-EGFR fluorescein isothiocyanate (FITC)-conjugated antibodies or anti-HER3 PE-conjugated antibodies (Santa Cruz Biotechnology Inc.) for 30 min on ice. Cells were washed and resuspended in 500 uL of flow buffer. Propidium iodide (PI) was added to each sample just before analysis on the cytometer. Samples were analyzed on a FACSCalibur flow cytometer (BD Biosciences, San Jose, CA, USA) and a minimum of 10,000 live events per sample was acquired. Histogram analysis was performed with FlowJo software (Tree Star Inc., Ashland, OR, USA). Data were restricted to live events based on PI exclusion.

### Phospho-kinase array

Ctx^R^ cell line (HC4) was analyzed in the panel of phosphorylation profiles of kinases after treatment with cetuximab, U3-1287 and a combination of cetuximab and U3-1287 agents (Human Phospho-Kinase Array, ARY003; R&D Systems). This array specifically screens for relative levels of phosphorylation of 46 individual proteins involved in cellular proliferation and survival. After 24 h of treatment with cetuximab (20 ug/mL), U3-1287 (100 ug/mL) and their combination, cells were harvested and cell lysates were incubated with the membrane. Thereafter, a cocktail of biotinylated detection antibodies, streptavidin–horseradish peroxidase and chemiluminescent detection reagents were used to detect the phosphorylated protein. The relative expression of specific phosphorylated protein was determined following quantification of scanned images by ImageJ compared with cetuximab, U3-1287, their combination and vehicle.

### Annexin-V apoptosis assay

An amount of 800,000 cells were plated in 100 mm plates and after 24 h of incubation treated with vehicle, 20 ug/mL cetuximab, 50 ug/mL U3-1287 or the combination for 24 h. Cells were harvested after trypsinization. Next, cells were washed with PBS, re-suspended in binding buffer (BD Biosciences) and stained with FITC Annexin-V (FITC Annexin-V apoptosis detection kit, BD Biosciences). The cells were analyzed by flow cytometry (BD FACScan). FlowJo Software (Tree Star, Inc.) was used to analyze the data. All experimental arms were done in triplicate and displayed as averages with standard error bars.

### Caspase 3/7 activity assay

Caspase 3/7 activity assay was performed by manufacture’s protocol (Promega). Briefly, Ctx^R^ clones were plated in a white-walled 96-well plate. Cells were treated with vehicle, 20 ug/mL cetuximab, 50 ug/mL U3-1287 or the combination for 24 h. After adding 100 μL of Caspase 3/7 reagent and mix gently, the cells were incubate for 1 h at room temperature, and the luminescence of each sample was measured by luminometer (Enspire plate reader, Perkin Elmer, Waltham, MA). Caspase 3/7 assay was carried out in triplicate.

### Mouse cetuximab-resistant human tumor xenografts

Athymic nude mice (4- to 6-week old; male) were obtained from the Harlan Laboratories (Indianapolis, IN, USA). All animal procedures and maintenance were conducted in accordance with the institutional guidelines of the University of Wisconsin. Mice were injected with H226 (2x10^6^ cells) and tumors were allowed grow to 100 mm^3^. All mice were randomized to treatment or control groups and treated with 1 mg/mouse (40 mg/kg) of either Cetuximab or IgG intraperitoneally twice per week. Tumors were monitored for cetuximab resistance that was defined as marked tumor growth in the presence of continued cetuximab therapy. Once Ctx^R^ tumors reached a volume ~800 mm^3^, mice were grouped according to similar time points of resistance. At this point each mouse was treated with cetuximab, 500 ug of U3-1287 or the combination of cetuximab and U3-1287 intraperitoneally twice per week. Tumor volume measurements were evaluated by digital calipers and calculated by the formula (p)/6 x (large diameter) × (small diameter)^2^.

### Mouse tumor collection and protein isolation

Tumors were collected 24 hours after last treatment. Mice were sedated using isofluorane mixed with oxygen, which was administered until loss of consciousness. Mice were euthanized by cervical dislocation and tumors were promptly collected, washed in PBS, and frozen with isopentane on dry ice. Whole cell protein lysates from tumor samples were obtained with NP-40 lysis buffer, homogenized by 10 strokes in a tightly fitting Dounce homogenizer, and quantified. Protein quantitation and immunoblotting analysis were performed as stated above.

### Immunohistochemistry

Tumor tissue samples were collected from xenograft tumors. Tumor samples were fixed in 10% neutral buffered formalin and paraffin embedded. Sections were heated in 10 mM citrate buffer (pH6.0) for Ki67 and cleaved caspase-3 by Decloaking chamber. Samples were incubated with rabbit anti Ki67 (1:400) and rabbit anti cleaved caspase-3 (1:200). Sections were stained by the Universal Quick kit (Vector laboratories, Inc., PK-8800, Burlingame, CA, USA). Antibody binding was revealed by addition of 3,3′-diaminobenzidine substrate (Thermo Fisher). Tissues were counterstained with Mayer’s hematoxylin (Thermo Fisher). Tissues were examined using an Olympus BX51 microscope. Quantitation of staining intensity was performed with ImageJ.

### Statistical analysis

Student t-tests were used to evaluate the significance of changes in all expression assays compared to non-targeting controls. Differences were considered statistically significant if P ≤ 0.05.

## Abbreviations

CRC: Colorectal cancer
CtxR: Cetuximab-resistant
CtxS: Cetuximab-sensitive
EGFR: Epidermal growth factor receptor
FDA: Food and Drug Administration
FITC: Fluorescein isothiocyanate
HP: Parental H226 cells
HNSCC: Head and neck squamous cell carcinoma
IP: Intraperitoneal
IgG: Immunoglobulin G
MAPK: Mitogen-activated protein kinase
mAb: Monoclonal antibody
NRG1: Neuregulin 1
NSCLC: Non-small cell lung cancer
RTK: Receptor tyrosine kinases
qPCR: Quantitative PCR
siRNA: Small interfering RNA
PI3K: Phosphatidylinositol 3-kinase.
